# Supplementary material for: Gossamer: Scaling Image Processing and Reconstruction to Whole Brains
Source: bioRxiv. 2024 May 29:2024.04.07.588466. Originally published 2024 Apr 9. Preprint. [Version 2] doi: 10.1101/2024.04.07.588466 (PMC11030332; doi:10.1101/2024.04.07.588466)
Supplement: Supplement 1 [file NIHPP2024.04.07.588466v2-supplement-1.pdf]

VII. Supplementary Material

Supplementary Note 1 *PyStripe:* To better eliminate CMOS camera artifacts, we modified the PyStripe program to apply a small 5x5 Gaussian kernel with a sigma of 1 to raw images before applying the destripe algorithm. Empirically, the db9 wavelet and a sigma of 250 for 15x images and

bioRxiv preprint doi: https://doi.org/10.1101/2024.04.07.588466; this version posted May 29, 2024. The copyright holder for this preprint (which was not certified by peer review) is the author/funder, who has granted bioRxiv a license to display the preprint in perpetuity. It is made available under aCC-BY 4.0 International license.

100 for 4x images for foreground and background produced better results. Since the original PyStripe algorithm was adding artifacts to the edges of the image, we temporarily padded the images with the wrap method before destriping. The pad size is automatically calculated based on the destriping sigma. Not the pad size is equal to the half-height width of the Gaussian notch filter.

We implemented a more efficient queue-based parallel processing model for pystripe and added PNG reading and damaged-image-detection-and-replacement functionality. Queue-based parallel processing model in contrast to map-function-based is more efficient since there is no need to spawn a new worker for each image. Instead, each worker runs only one time and processes a queue of images. The queue-based model is specifically more beneficial on Windows, which has more overhead to spawn workers compared with Linux which forks them most of the time.

Using Numba[24] and NumExpr [15], we optimized the pipeline by the just-in-time compilation of some of the functions where beneficial. Also, we implemented a fast blank image detection algorithm to skip the processing of such images to further speed up the process.

Supplementary Note 2 *Parastitcher:* We implemented a multi-GPU accelerated and NAS-friendly version of Parastitcher[10] on Linux to find the alignment of tiles. We patched TeraStitcher to repeat the read request up to 40 times to fix an issue with some NAS storage in which the reading request fails sometimes.

Supplementary Note 3 *TSV:* We also employed a queue-based parallel processing model for the TeraStitcher volume program[39] that is more resilient to missing tiles. We increased the resiliency of TSV in case of missing tiles; TSV can now detect missing tiles and replace them with blank images. We also made TSV compatible with newer versions of Python (v3.11 at the time of publication).

Supplementary Note 4 *16-bit to 8-bit conversion with variable bit shifting:* lightsheet fluorescent data has an unsigned 16-bit int type and brightness values have a gamma distribution. Traditional 8-bit conversion will shift the bits to the right for 8 bits, which assigns any value between 0 to 255 to zero while over-representing the tail of the gamma distribution. It is possible to clip the tail of the data so that the 16-bit image can be shifted to the right with a smaller number of bits, which leads to the assignment of a narrower range to zero. For example, with 1 right bitshift only 0 to 1 will be assigned to zero in 8-bit. We used a multi-class Otsu thresholding function from the scikit-image package[40] with 4 classes that are applied to the log1p transform of the image. Then we found 99.9 percentile of values larger than the upper threshold to find the upper bound clipping value. For the lower bound of the data, we made sure any value larger than one that could be set to zero by bit-shifting was assigned to 1 instead.

Supplementary Note 4 *Non-blocking Paraconverter:* We used Paraconverter to convert stitched images to TeraFly format[11]. Since Paraconverter did not scale well on our cluster computer, we implemented a parallel processing algorithm that runs Paraconverter without blocking the progress of stitching for the remaining channels. We used TeraFly files for seed detection, visualization of the images in virtual reality (TeraVR)[42], and vetting of neuronal reconstruction.

Supplementary Note 5 *Multi-channel images:* we developed an algorithm that finds the translation of the stitched images for a reference channel (usually the nuclear staining channel that is used for image registration to 3D brain atlases) at the middle z-frame of the volumes using the OpenCV version of enhanced correlation coefficient maximization algorithm[17] before converting the images to RGB format. Finally, the 2D RGB tif series are converted to a single Imaris volume with a custom code in Python.

Supplementary Note 6 *Automation:* We minimized human interaction by automatically calculating the number of processes based on the required RAM and the available resources. Also, we made sure the RAM usage was optimal by avoiding declaring unnecessary temporary variables since the size of each z-step of the stitched images can be in the order of several GBs in RAM.

Supplementary Note 7 *DeBleach:* `coif15` wavelet is used for initial bidirectional destriping. We used sosfiltfilt function from SciPy Signal package[33] with a frequency of (1/tile size) for generating the low-pass filter because it has no phase shift. The foreground of the image was clipped to the max of the background in advance.

Supplementary Note 8 *Deconvolution:* We optimized the deconvolution algorithm to minimize copying data back and forth between system RAM and video RAM (vRAM), which enhanced the performance significantly. We also optimized block size calculation to ensure the maximum block size on vRAM so that larger chunks of the image volume can be processed at any given time. Image blocks now load asynchronously in the background to maximize GPU utilization time. We also fixed a bug in the program, which was adding artifacts on the z-axis, by padding image blocks using the data from the image volume instead of an artificial pad on the z-axis.

For an unknown reason, some of the lightsheet images could show a duplication effect on the z-axis, or neurites could look like halo tubes, which cause problems for reconstruction. Empirically, we found that a 3D Gaussian filter with sigmas of [0.5, 0.5, 1.0] and a kernel size of [3, 3, 9] on x, y, and z axes respectively, can mitigate the issue post hoc. Deconvolution algorithms are sensitive to noise. 3D Gaussian filter can also further denoise the images to improve deconvolution.
